# Optical mapping of the pig heart *in situ* under artificial blood circulation

**DOI:** 10.1038/s41598-020-65464-5

**Published:** 2020-05-22

**Authors:** Irma Martišienė, Dainius Karčiauskas, Antanas Navalinskas, Regina Mačianskienė, Audrius Kučinskas, Rimantas Treinys, Ramunė Grigalevičiūtė, Vilma Zigmantaitė, Laima Ralienė, Rimantas Benetis, Jonas Jurevičius

**Affiliations:** 1 Institute of Cardiology, Lithuanian University of Health Sciences, Kaunas, Lithuania; 2 Department of Cardiac, Thoracic and Vascular Surgery, Hospital of Lithuanian University of Health Sciences Kauno Klinikos, Lithuanian University of Health Sciences, Kaunas, Lithuania; 3 Biological Research Centre, Lithuanian University of Health Sciences, Kaunas, Lithuania

**Keywords:** Biological fluorescence, Ventricular tachycardia

## Abstract

The emergence of optical imaging has revolutionized the investigation of cardiac electrical activity and associated disorders in various cardiac pathologies. The electrical signals of the heart and the propagation pathways are crucial for elucidating the mechanisms of various cardiac pathological conditions, including arrhythmia. The synthesis of near-infrared voltage-sensitive dyes and the voltage sensitivity of the FDA-approved dye Cardiogreen have increased the importance of optical mapping (OM) as a prospective tool in clinical practice. We aimed to develop a method for the high-spatiotemporal-resolution OM of the large animal hearts *in situ* using di-4-ANBDQBS and Cardiogreen under patho/physiological conditions. OM was adapted to monitor cardiac electrical behaviour in an open-chest pig heart model with physiological or artificial blood circulation. We detail the methods and display the OM data obtained using di-4-ANBDQBS and Cardiogreen. Activation time, action potential duration, repolarization time and conduction velocity maps were constructed. The technique was applied to track cardiac electrical activity during regional ischaemia and arrhythmia. Our study is the first to apply high-spatiotemporal-resolution OM in the pig heart *in situ* to record cardiac electrical activity qualitatively under artificial blood perfusion. The use of an FDA-approved voltage-sensitive dye and artificial blood perfusion in a swine model, which is generally accepted as a valuable pre-clinical model, demonstrates the promise of OM for clinical application.

## Introduction

The emergence of optical imaging has undoubtedly revolutionized the investigation of cardiac electrical activity^1,2^. The electrical signals in the heart and the associated propagation pathways are crucial for elucidating the mechanisms of various cardiac pathological conditions, including arrhythmia. The first heart activation maps were constructed based on electrical signals obtained by arrays of electrodes^3,4^ or optical fibres^5^ from hundreds of recording sites separated by a few millimetres. Because of the limited number of points, such techniques have low resolution. Meanwhile, the main characteristic of current optical mapping (OM) methods is high spatiotemporal resolution^1,6^. Because of this property, OM with voltage-sensitive dyes is widely used in the investigation of cardiac arrhythmogenesis at the cellular, tissue and organ levels. Isolation of the heart from an organism has been demonstrated to influence the mechanisms of arrhythmia formation^7^. Therefore, *in situ* investigations are of particular importance for identifying mechanisms similar to those that occur in nature during pathophysiological conditions. The synthesis of near-infrared (NIR) voltage-sensitive dyes that can be used in blood-perfused hearts^8^ and the demonstration of the voltage sensitivity of the FDA-approved dye Cardiogreen^9–11^ have increased the potential of OM as a prospective useful tool in clinical practice.

The first attempt to apply OM *in situ* was reported by Lee *et al*.^12^, who developed a cardiopulmonary bypass circuit in rats, recorded voltage and calcium signals, and demonstrated the feasibility of the multiparametric imaging of a mammalian heart *in vivo*. However, due to the challenging procedures and problems associated with motion artefacts in live organisms, the use of high-spatiotemporal-resolution OM for large animal hearts *in situ* has not been reported for a long time since the OM technique was developed. Moreover, the emergence of studies investigating the impact of autonomic nervous regulation on arrhythmia formation in the intact heart^13,14^ have encouraged the development of OM systems adapted for large animals/humans *in situ*. The swine model is generally accepted as a valuable pre-clinical model because of its large heart and body weight and the similarity of its cardiovascular system to that of humans^15,16^. Recently, OM was performed in freely contracting pig hearts^17^. The authors used an optical fibre array and recorded optical signals (OSs) from 16 locations on the pig heart and also performed a ratiometric analysis in the whole heart. Using excitation ratiometry, the electrical activity of the heart was recorded during the application of a high stimulation frequency or during fibrillation, i.e., the conditions when the impact of contraction on the OS is at least partially suppressed. Despite this, the maps of various action potential (AP) parameters were not provided, suggesting that their technique lacks one of the most important OM features, i.e., registration at high spatial resolution. Evidently, excitation ratiometry helps to minimize contraction artefacts. However, comprehensive studies have demonstrated that additional mechanical heart immobilization or mathematical ascertainment of the pixel and heart surface are required to use ratiometric OM in freely contracting hearts^6,18^.

Our aim was to develop an OM system adapted for large animals and suitable for the high-spatiotemporal-resolution recording of cardiac electrical activity under artificial blood perfusion, a condition that is widely used in cardiac surgery. Additional experiments were performed in freely contracting heart using glued metal frame for the immobilization of a part of anterior surface of the heart under physiological blood circulation. We used a swine model and performed open-chest heart surgery. Using the fluorescent, voltage-sensitive dyes di-4-ANBDQBS and Cardiogreen, we recorded the electrical activity of the heart *in situ*. In this paper, we present a detailed description of the methods used and high-spatiotemporal-resolution OM data obtained from pig hearts *in situ* under normal and pathophysiological conditions. The use of artificial blood perfusion and an FDA-approved voltage-sensitive dye demonstrates the promise of the registration of cardiac electrical activity for clinical application, such as in open-heart surgery.

## Results

### Optical mapping of the pig heart *in situ* under physiological blood circulation using fluorescent di-4-ANBDQBS dye

**Figure 1:**
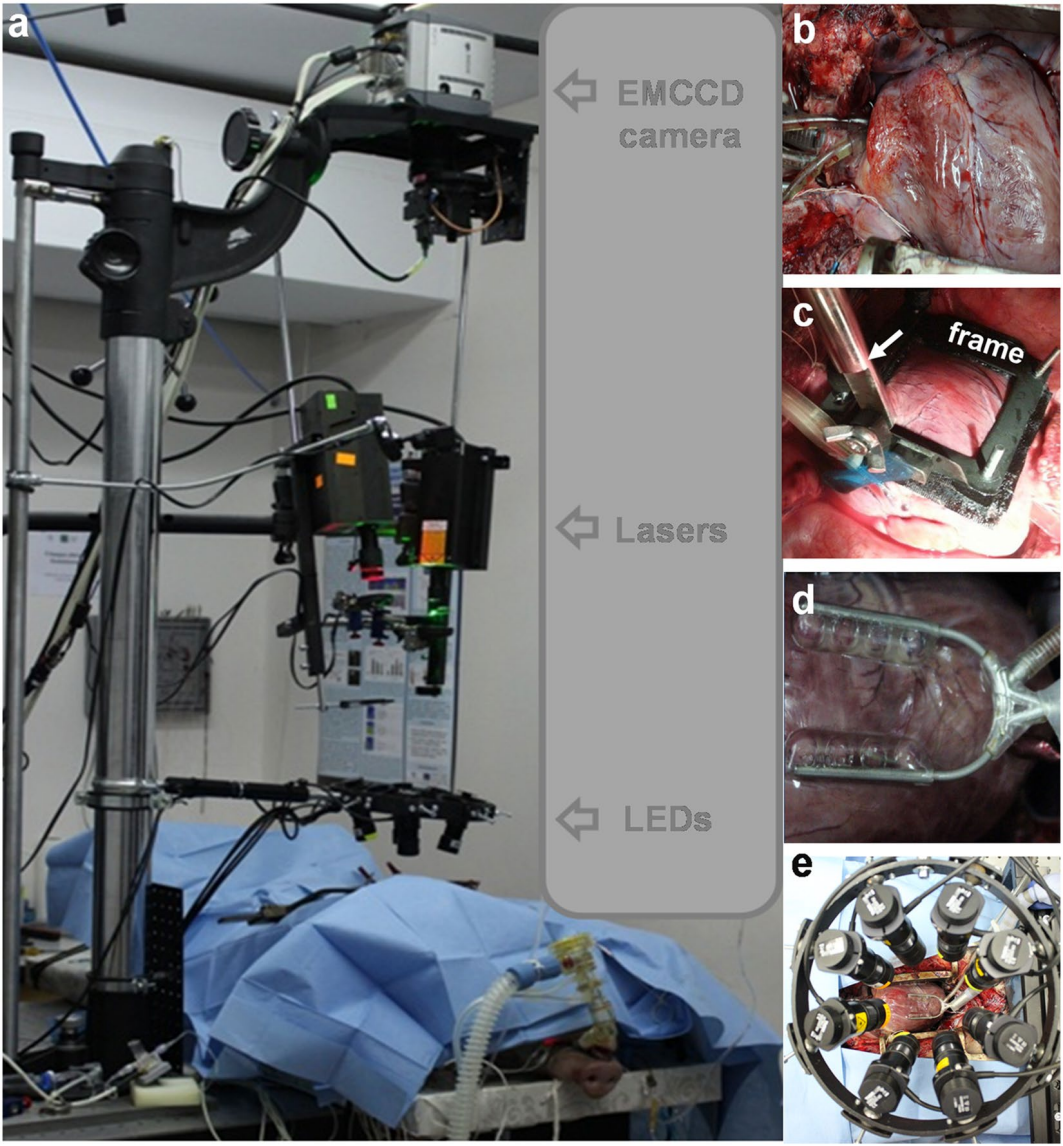
Overall setup for optical mapping of the whole pig heart *in situ*. (**a**) Whole optical mapping setup adapted for registration of the cardiac electrical activity of the pig heart *in situ* during open-heart surgery. (**b**) Heart with inserted cannulas. (**c**) Hand-made frame for heart immobilization. Arrow indicates the bar. (**d**) Octopus tissue stabilizer used for heart immobilization. (**e**) Wheel with 8 positions for excitation LEDs (four 660 nm and four 780 nm).

First, we attempted to register the electrical activity of the pig heart under physiological blood circulation using different heart immobilization methods. We performed experiments using a hand-made frame (Fig. 1c) or a clinically used tissue stabilizer (Fig. 1d) without the administration of chemical electromechanical uncouplers and using the dye di-4-ANBDQBS.

**Figure 2:**
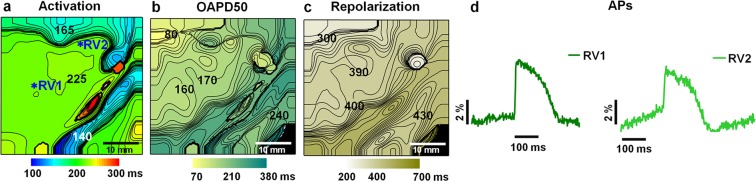
Electrical activity in the pig heart immobilized by the frame *in situ* and recorded by optical mapping using di-4-ANBDQBS under physiological blood circulation. Atrial pacing with a 500-ms period was applied. The metal frame was fixed to the heart surface, mostly on the right ventricle. Maps of the activation time, OAPD50 and repolarization time are presented in (**a–c**). The numbers near the isochrones show the activation time, OAPD50 and repolarization time in ms. The interval between isochrones is 5 ms for the activation time and 10 ms for the OAPD50 and repolarization time. Optical action potentials (OAPs) were obtained from 5 × 5 pixels at different locations of the right ventricle (RV1, RV2), as indicated by asterisks in map (**a**) and presented in (**d**). The amplitude of the OAPs is given as a percentage with respect to the background (ΔF/F). The mapping area was 40 × 40 mm.

The results of the OM of the pig heart immobilized by the frame are presented in Fig. 2 (see also *Online Supplementary Material*, Movie S1, Fig. S5a). The activation time, optical action potential duration at 50% and at 80% repolarization (OAPD50, OAPD80, respectively), and repolarization time maps show that APs could be registered by this method, although the quality of the APs was restricted in terms of area because at the edges of the mapping area, the OAPs were distorted by contractions (Fig. 2d RV1 *vs*. RV2). The coronary arteries also acted as a morphological obstacle that significantly reduced the intensity of the OSs from this area. The maximal fractional change in the fluorescence signal (ΔF/F) was 6.1 ± 1.8% (n = 5), on average.

Similar optical data were obtained using an Octopus tissue stabilizer (data not shown).

Due to the low quality of the recordings, we did not continue investigations of OM *in situ* under physiological blood circulation.

### Optical mapping of the pig heart *in situ* under artificial blood circulation using fluorescent di-4-ANBDQBS dye

In experiments with artificial blood circulation using di-4-ANBDQBS, the ΔF/F was 16.17 ± 2.08% (n = 4). Representative recordings of the optical signals obtained during ventricular pacing with a 500-ms period under the control are presented in Fig. S6. The relatively high optical signal encouraged us to perform registrations of electrical activity under different conditions that may be useful in applying OM in order to investigate certain patho/physiological cardiac mechanisms in the conditions of the whole organism.

**Figure 3:**
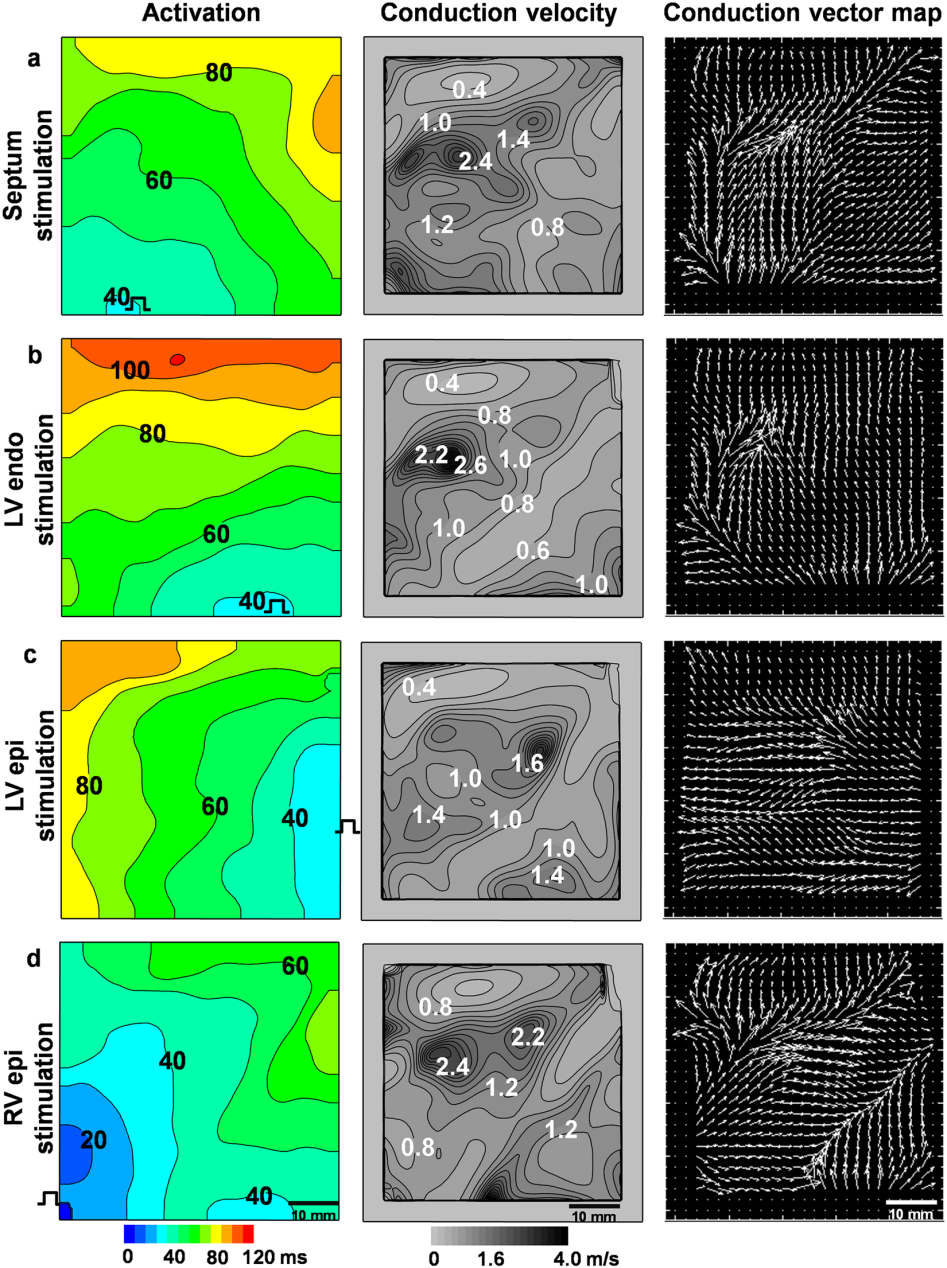
Electrical activity in the pig heart recorded by optical mapping *in situ* using the fluorescent dye di-4-ANBDQBS under artificial blood circulation. Activation time, conduction velocity and vector maps were obtained during pacing at four different locations, i.e., the septum (**a**), left ventricular endocardium (LV endo) (**b**), left ventricular epicardium (LV epi) (**c**), and right ventricular epicardium (RV epi) (**d**), with a 500-ms period. The numbers near the isochrones show the activation time in ms in the activation maps and the conduction velocity in m/s in the conduction velocity maps. The interval between isochrones is 10 ms for the activation time and 0.2 m/s for the conduction velocity.

Figure 3 shows the activation time, conduction velocity and vector maps obtained during pacing at four different locations, i.e., the septum, left ventricular endocardium, left ventricular epicardium, and right ventricle (RV), over a 500-ms period. The data clearly demonstrate that OSs obtained during OM of the pig heart *in situ* under artificial blood perfusion can be used to evaluate the activation time, the direction of excitation propagation and the conduction velocity. The average conduction velocity independent of stimulation location was higher in the RV than in the left ventricle (LV), i.e., 1.21 ± 0.13 m/s *vs*. 0.91 ± 0.1 m/s (n = 4, p < 0.05).

In addition to stimulation from different heart locations, various pacing cycle periods were applied. Normalized OAPs obtained under different stimulation periods (200–500 ms) and excitation wavelengths (green, red), as well as OAPs calculated as the ratio of the OS under green and red light excitation, are presented in the *Online Supplementary Material* (Fig. S2). Movies showing the propagation of excitation of the pig heart under all conditions are presented in the *Online Supplementary Material* (Movies S2–S6). OAP distortion potentially caused by residual motion was apparent under all applied registration conditions. Slightly greater OAP distortion was detected under green light excitation because the ΔF/F of the OSs under green light was smaller than that under red light. In our experiments, the ratio of the two signals, which is commonly determined during OM to remove motion artefacts^19,20^, did not obviously differ from the OAPs obtained under red light excitation. The data show that the OAP alternans appeared during the pacing period of 250–200 ms under green light but were not apparent under red light (Fig. S2a *vs. b*). Under different excitation wavelengths, OAPs originate from different myocardial depths^21,22^. In our presented example, the OAP alternans were recorded in the epicardial layer of the left myocardium. Meanwhile, the results show that the ratio barely reflects the alternans. Thus, fluorescence ratiometry should be used with caution in arrhythmia investigations performed to detect the differences between myocardial layers that may underlie the mechanisms of various arrhythmias.

Because green light penetrates the myocardium less than red light, we examined the upstroke of OAPs using different excitation wavelengths. The OAPs obtained under green and red excitation and their ratios at normal and magnified time scales are presented in the *Online Supplementary Material* (Fig. S4). The OAPs from the right and left ventricles showed no significant difference in the OAPD; however, the OAP upstroke duration was less under green than red excitation. These data support our previous data obtained from a Langendorff perfusion rabbit heart model^21^. Moreover, the results show that in the case of the LV, which is thicker than the RV, the ratio of the collective signals hides differences in the OAP upstroke duration at different myocardial layers. This again suggests that fluorescence ratiometry should be used with caution when evaluating the OAP upstroke, which often serves as a sensitive indicator of various pathologies in cardiac electrophysiology.

### Correlation between OAPs and electrical signals

**Figure 4:**
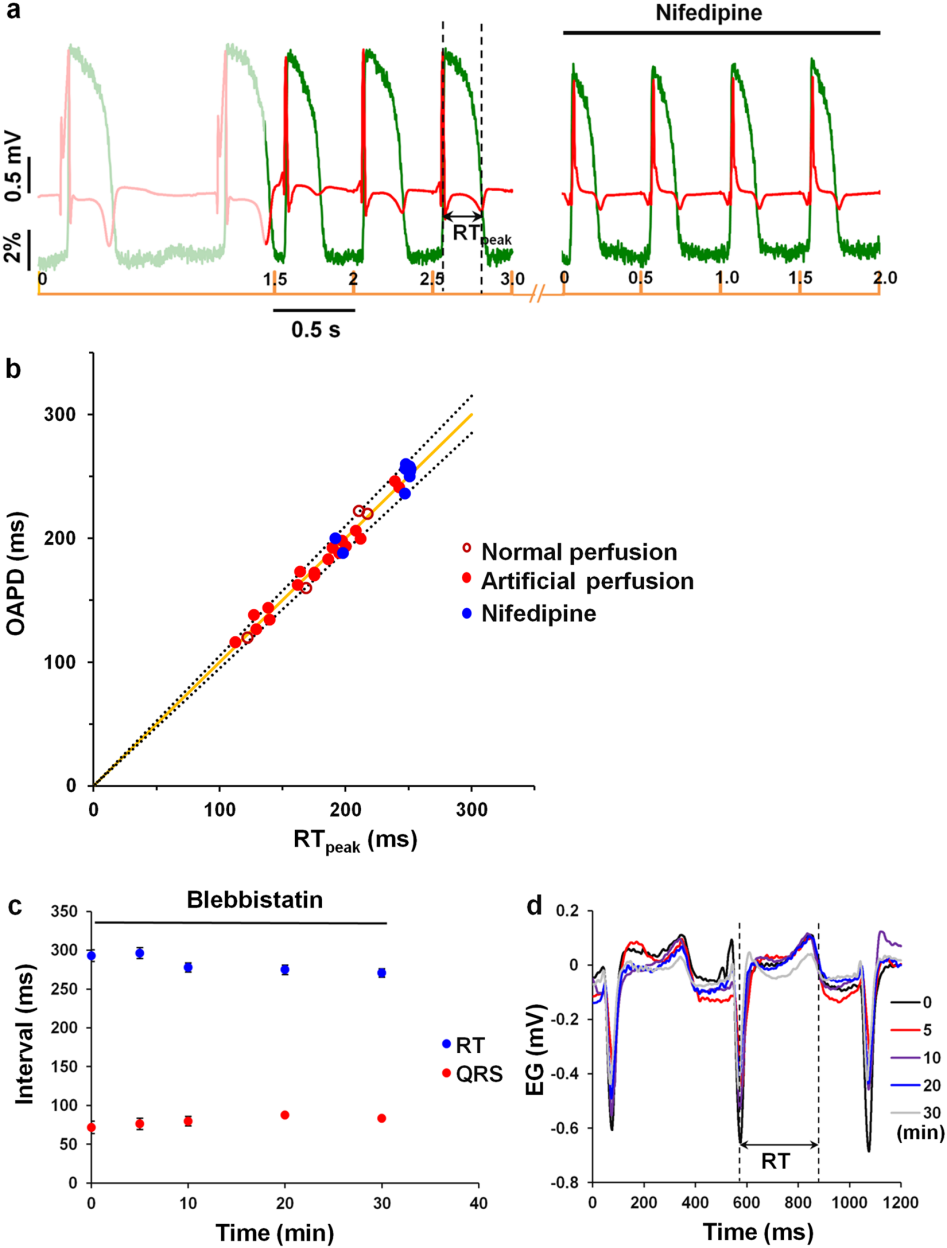
Correlation between the optical and electrical signals of the pig heart. Optical signals were recorded by optical mapping *in situ* using di-4-ANBDQBS and electrical signals via electrography under artificial blood circulation. (**a**) Experimental recordings of optical action potentials obtained using di-4-ANBDQBS and electrograms during spontaneous rhythm and under applied stimulation with a period of 500 ms under the control condition and after the injection of a 50-mL bolus containing nifedipine (100 µmol/L). (**b**) The correlation between the duration of the optical signal at 60% repolarization (OAPD) and electrical signal (RT_peak_) obtained under normal-physiological (n = 4, open red dots) or artificial blood perfusion, including with nifedipine (n = 4, closed red and blue dots, respectively). The presented data were obtained during spontaneous activity and a pacing period of 200–500 ms. The solid line indicates the theoretical linear dependence of OAPD and RT_peak_, and the dashed lines indicate the boundaries of 5% deviation from the absolute values of the line points. (**c**) Effect of blebbistatin (bolus concentration 600 µmol/L) on the RT (blue points) and QRS (red points) intervals (n = 4). The pacing period was 500 ms. (**d**) Overlapped electrogram fragments recorded at different times after exposure to blebbistatin. The dashed lines mark the boundaries of the RT interval. EG, electrogram.

Sufficient data are available to show the direct correlation between OAPs and electrical signals^1^. To validate the optically determined signals obtained from the pig heart *in situ*, the unipolar electrogram was recorded simultaneously with optical recordings. Figure 4a shows experimental recordings of OAPs obtained using di-4-ANBDQBS and electrograms during spontaneous rhythm and under an applied stimulation with a period of 500 ms. Additionally, the L-type Ca^2+^ channel inhibitor nifedipine, which shortens the AP duration, was applied as a bolus. The data show that an OS under our experimental conditions follows the changes in the electrogram depending on the heart rate and/or on chemical modification. Figure 4b shows the correlation between the duration of optical and electrical signals obtained under normal (n = 4, open red dots) or artificial blood perfusion, including with nifedipine (n = 4, closed red and blue dots, respectively) during spontaneous activity and the application of pacing over a period of 200–500 ms. It is commonly known that the QT interval reflects the AP duration^23^. In our case, a unipolar electrogram with variable wave polarity in different experiments was recorded. To obtain the most accurate values, the duration of the electrical signal was evaluated as the RT interval measured from the peak of the R wave to the peak of the T wave (RT_peak_, indicated by the dashed line in Fig. 4a). The ratio of the optical and electrical signal durations was obviously linear (solid line indicates theoretical linear dependence), and the distribution of the experimental points was fit within a 5% deviation from the absolute values of the theoretical line points (dashed lines) with a high average correlation coefficient of 0.985 ± 0.005 (n = 5). For the calculation of the average correlation coefficient, five coefficients were included. One coefficient was obtained from the data collected from four experiments under normal blood perfusion. The other four coefficients were obtained from the data of four different pig experiments with artificial blood perfusion. The best correlation between the optical and electrical signals was obtained using the OAPD at 60% repolarization. The correlation coefficient using the OAPD at 90% repolarization was not assessed because at this level, the OS was sensitive to residual motion artefacts.

### Effect of blebbistatin on electrical activity of pig heart recorded under artificial blood perfusion *in situ*

In our experiments, blebbistatin was used under artificial blood perfusion and injected as a bolus into the coronary system together with fluorescent dye. The wide use of this uncoupler in OM is based on investigations showing no significant effect on cardiac electrical activity^24^. However, other authors have reported the significant effect of blebbistatin on the electrical activity of isolated heart^17,25^. Therefore, we aimed to evaluate the effect of blebbistatin on the electrical parameters of the pig heart *in situ*. The RT and QRS intervals were measured to evaluate the APD and conduction velocity, respectively. Figure 4c shows the changes in the electrical signal after the injection of a 100-mL bolus containing 600 µmol/L blebbistatin. Figure 4d shows representative electrogram recordings indicating the RT interval measurement. In this case, the end of the T wave was considered the point of the maximal rate of descent of the T wave (RT, dashed line). The data show that the effect of blebbistatin on the duration of the RT and QRS intervals was not statistically significant. Before and 30 min after blebbistatin administration, the RT interval was 292.89 ± 7.5 ms and 270.6 ± 5.46 ms, and the QRS interval was 71.85 ± 8.12 ms and 83.33 ± 1.69 ms (n = 4, p > 0.05), respectively. It should be noted that the same bolus contained the fluorescent dye; however, recent data have demonstrated that di-4-ANBDQBS is not toxic to pig vital organs^17^.

In our experiments, the dose of blebbistatin up to 15 µmol/L (recalculated in circulating blood), that is generally accepted to stop cardiac contractions, sometimes did not sufficiently suppress the motion. Moreover, a while after blebbistatin injection, contractions were partly restored, which was possibly related to metabolism of the drug; therefore, the same dose of uncoupler was injected again. In some experiments, diltiazem was also applied, which fully stopped the motion and prevented ventricular arrhythmia.

### Optical mapping of the pig heart *in situ* under artificial blood circulation using the fluorescent dye Cardiogreen

**Figure 5:**
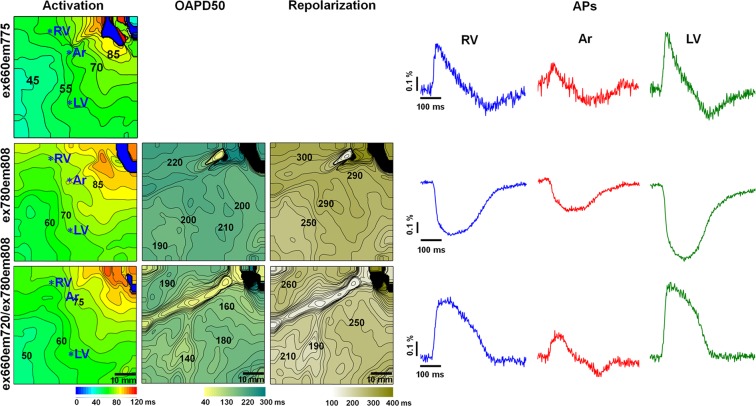
Electrical activity in the pig heart *in situ* recorded by optical mapping using the fluorescent dye Cardiogreen at various excitation and emission wavelengths during apex pacing with a 500-ms period under artificial blood circulation. The maps of the activation time and OAPs from the sites indicated by asterisks in the activation map in the regions of the right ventricle (RV), artery (Ar), and left ventricle (LV), obtained at λ_ex_ = 660 nm and λ_em_ = 775 nm, are presented in the *top row*. The maps of the activation time, OAPD50 and repolarization time and the OAPs from the sites indicated by asterisks in the activation maps obtained at λ_ex_ = 780 nm and λ_em_ = 808 nm are presented in the *middle row*. The activation time, OAPD50 and repolarization time maps and the OAPs constructed from the OAPs calculated as the ratio of the two OSs obtained at λ_ex_ = 660 nm/λ_em_ = 720 nm and λ_ex_ = 780 nm/λ_em_ = 808 nm are presented in the *bottom row*. The numbers near the isochrones show the activation time, OAPD50 and repolarization time in ms. The interval between isochrones is 5 ms for the activation time and 10 ms for the OAPD50 and repolarization time. The amplitude of the OAPs is given as a percentage with respect to the background (ΔF/F).

In the next set of experiments on recording the electrical activity of the pig heart *in situ*, we used the dye Cardiogreen. Previously, we showed that the OS obtained by Cardiogreen is composed of two components, fast and slow, and that the characteristics are dependent on the excitation/emission wavelengths (λ_ex_ and λ_em_)^10,11^. To demonstrate that Cardiogreen can be used for the OM of the heart *in situ*, we applied three optimal excitation/emission conditions suitable for recording cardiac electrical activity, i.e., λ_ex_ = 660 nm/λ_em_ = 775 nm, λ_ex_ = 660 nm/λ_em_ = 720 nm, and λ_ex_ = 780 nm/λ_em_ = 808 nm. Representative results showing the activation time, OAPD50 and repolarization time maps and the OAPs from the RV, artery (Ar), and LV, indicated by asterisks in the activation maps obtained at various λ_ex_ and λ_em_ values, are presented in Fig. 5. At λ_ex_ = 660 nm and λ_em_ = 775 nm, positive OSs were obtained^11^. At such λ_ex_/λ_em_ values, the OS of Cardiogreen consisted mainly of the fast component, and the OAP exhibited a characteristic steep upstroke, which allowed for the construction of an activation time map (Fig. 5*, top row*). The repolarization phase of the OS was clearly distorted; therefore, construction of the OAPD map was irrelevant. The OS recorded at λ_ex_ = 780 nm and λ_em_ = 808 nm included two components, which were both negative (Fig. 5, *middle row*). The presence of the slow component makes the upstroke of the OS less steep than that of the OS obtained at λ_ex_ = 660 nm, and the signal has a longer duration than the AP of a single cell. Notwithstanding, the signals were sufficient to construct activation, OAPD50 and repolarization time maps (Fig. 5*, middle row*, for OAPD80 map see *Online Supplementary Material* Fig. S5), which allow for the qualitative evaluation of the propagation and distribution of OAPD and repolarization time. To approximate the OS recorded by Cardiogreen for quantitative evaluation, use of the relation of the two signals obtained at λ_ex_ = 660 nm/λ_em_ = 720 nm and λ_ex_ = 780 nm/λ_em_ = 808 nm is recommended^10,11^. The results of the relation of the two signals are presented in the bottom row of Fig. 5. The OS from the region under the coronary arteries is clearly weaker, as shown in the experiments using di-4-ANBDQBS. Therefore, the OAPD50 map constructed from the OAPs calculated as a relation between the two signals obtained at different λ_ex_/λ_em_ values shows very short OAPD50 values in the region of the coronary arteries (Fig. 5*, bottom row*). OSs recorded at λ_ex_ = 660 nm/λ_em_ = 720 nm were used only for the calculation of the ratio and are not presented.

Thus, the results show that the FDA-approved dye Cardiogreen, which is clinically used for vessel visualization, can be used for the registration of cardiac electrical activity in the pig heart *in situ*. Recordings of cardiac electrical activity composed of OS relations obtained by Cardiogreen in the pig heart are presented in Movie S7 (*Online Supplementary Material*).

### Comparison of optical signals recorded by di-4-ANBDQBS and Cardiogreen under artificial blood circulation *in situ*

To compare the OSs obtained by the fluorescent dyes di-4-ANBDQBS and Cardiogreen, parameters, such as the maximal ΔF/F, maximal signal-to-noise ratio (SNR) and SNR decay rate, were evaluated. The parameters were calculated from the OSs obtained ~15 min after dye injection. The average values are presented in Table 1. Note that the OS for calculating the SNR was not averaged. All the parameters given for Cardiogreen were calculated from the signal ratio, i.e., λ_ex_ = 660 nm/λ_em_ = 720 nm and λ_ex_ = 780 nm/λ_em_ = 808 nm.

**Table 1:** Parameters of the optical signals of di-4-ANBDQBS and Cardiogreen obtained under artificial blood circulation in the pig heart *in situ*.

	di-4-ANBDQBS n = 4	Cardiogreen n = 4
ΔF/F (%)	16.17 ± 2.08*	0.41 ± 0.12
SNR (dB)	33.5 ± 1.5*	26.5 ± 2.5
SNR decay rate (min^−1^)	0.027 ± 0.002*	0.049 ± 0.009

ΔF/F, maximal fractional change in the fluorescence signal; SNR, signal-to-noise ratio; *p < 0.05 *vs*. Cardiogreen.

The data show that the maximal ΔF/F and SNR were significantly lower and the SNR decay rate was higher for Cardiogreen than for di-4-ANBDQBS.

### Optical mapping of the pig heart *in situ* using fluorescent, voltage-sensitive dyes in pathological situations

The data above clearly show the capability of using OM for the registration of myocardial electrical activity *in situ*. Furthermore, we attempted to evaluate the applicability of this method *in situ* during various pathological conditions.

#### Regional ischaemia

In some experiments, acute regional ischaemia was induced by occlusion of the left anterior descending coronary artery by encircling it with surgical sutures. The experiments were performed under artificial blood circulation using di-4-ANBDQBS. The activation and OAPD maps, which are presented in the *Online Supplementary Material* in Fig. S6a–d, show a typical response to ischaemia, i.e., the formation of an ischaemic zone with slowed propagation and shortened OAPs. Correspondingly, the amplitude and duration of OAPs in the RV, LV and Ar from the ischaemic zone were reduced, while the OAPs from the non-ischaemic zone remained the same as before artery occlusion (*Online Supplementary Material*, Fig. S6, RV1, RV2, Ar1, Ar2, LV1, LV2 control (*e*) *vs*. ischaemia (*f*)).

#### Ventricular tachycardia/fibrillation

To evaluate the possibility of OM for recording arrhythmias *in situ*, ventricular tachycardia (VT) or ventricular fibrillation (VF) was induced by applying burst pacing with a frequency higher than 10 Hz. During VT/VF, reentrant-type disorders generally dominate. Usually, these disorders are related to the formation of two types of mother rotors, i.e., functional mother rotors, which appear to form because of the distribution of APDs and occur at different sites in the heart but are more common in the RV, and anatomical obstacles, which usually develop at sites where cardiac tissue, usually in the LV, is damaged by various pathological conditions^26,27^.

**Figure 6:**
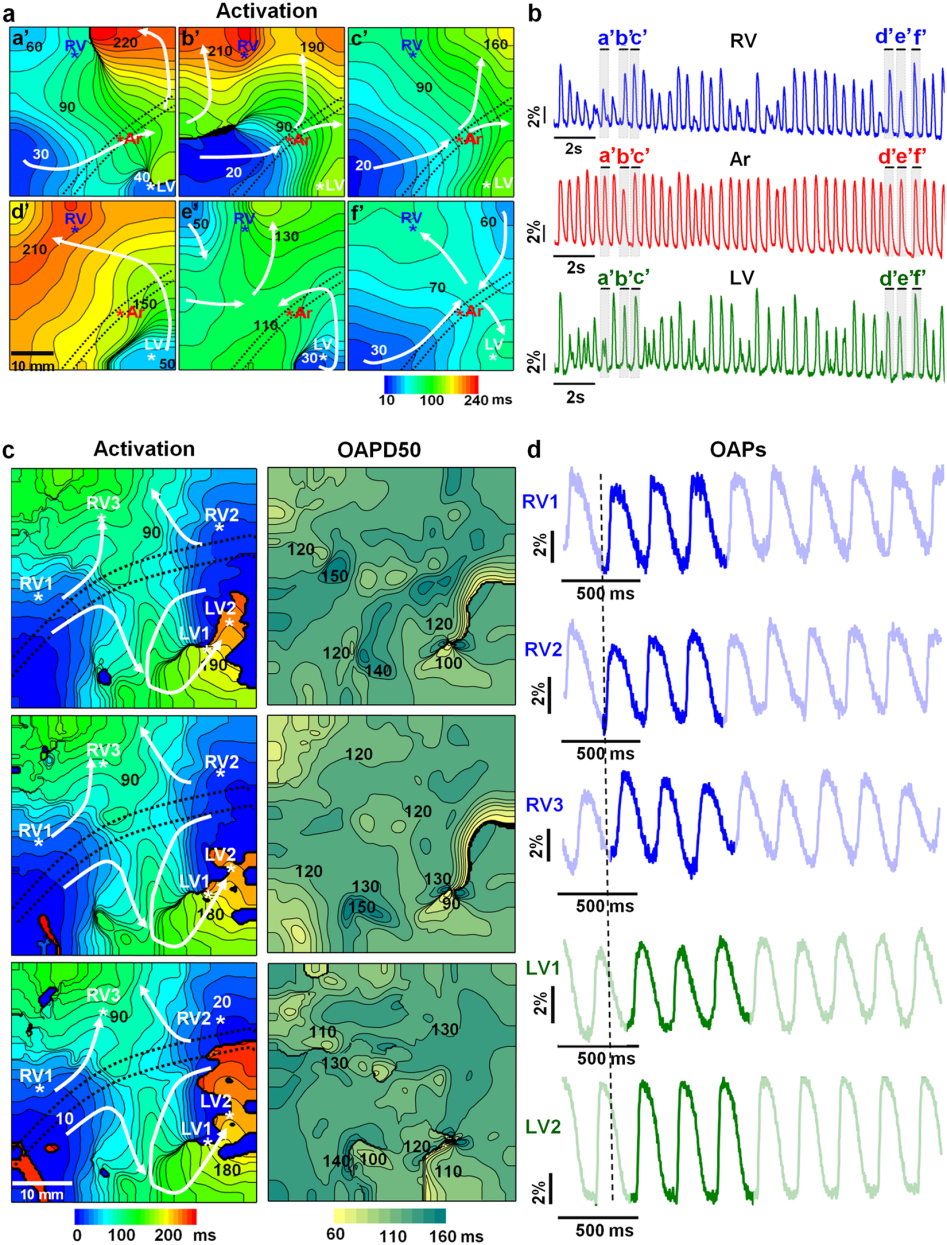
Ventricular reentrant arrhythmias with possible functional obstacles (**a,b**) and with possible stable anatomical obstacles (**c,d**) recorded by optical mapping of the pig heart *in situ* using di-4-ANBDQBS under physiological (**a,b**) or artificial blood circulation(**c,d**). (**a**) Activation time maps. The numbers near the isochrones show the activation time in ms. The interval between isochrones is 10 ms. The mapping area was 40 × 40 mm. The excitation period was approximately 500 ms (42 excitations per 20 s). The dominant frequency in the right ventricle (RV) was 9.3 Hz. (**b**) Recordings of raw optical traces from the regions of the RV, coronary artery (Ar), and left ventricle (LV), indicated by asterisks in the activation maps. The OAPs are labelled with letters (a’-f’) corresponding to the labelling of the activation time maps. (**c**) Activation time and OAPD50 maps (*left and right columns*, respectively). The numbers near the isochrones show the activation time and OAPD50 in ms. The interval between isochrones is 10 ms. The mapping area was 55 × 55 mm. The dominant frequency in the LV was 15.6 Hz. (**d**) Recordings of raw optical traces from the sites indicated by asterisks in the activation maps (**c***, left column*) from several regions of the RV and LV, starting from the vertical dashed line. The three bright OAPs (**d**) corresponding to the three OAPD50 maps. The white arrows indicate the direction of wave propagation. The black dotted lines on the maps indicate the central part of the anterior interventricular sulcus, where the coronary vessels are located. The amplitudes of the OAPs are given as percentages with respect to the background (ΔF/F).

In our experiments, we did not aim to investigate the mechanisms underlying reentrant arrhythmia development; however, OM of the pig heart *in situ* allowed us to register different types of wavefront circularities during VT/VF. An example of the multidirectional spread of excitation waves with different directions of propagation in every excitation cycle was obtained under physiological blood circulation using the hand-made frame for heart immobilization (Fig. 6a,b). The activation time maps (Fig. 6a) show the meandering wavefront (directions are indicated by white arrows). Raw recordings (20 s in duration) of optical traces obtained from the RV, Ar, and LV, indicated by asterisks on the activation maps, are presented in Fig. 6b. The OAPs are labelled with letters corresponding to the labels on the activation time maps. Note that the development of VT/VF lessens the influence of motion on the OS^28^. Raw optical traces show that the OAPs recorded in the coronary artery region are less distorted than those recorded in the ventricles. In other regions of the heart (RV and LV), multiple wave breakups occur, and the regularity of the signals is destroyed. The OSs were normalized to the background fluorescence. Therefore, if the whole ventricular wall was electrically excited, then the OSs from the same locations should be of the same amplitude. The smaller amplitudes of certain OAPs (a’, b’, e’, in the RV and LV, Fig. 6b) indicate that not all myocardial cells were transmurally activated. This finding suggests that we observed 3D scroll waves. The possible intramural propagation of the excitation wave obtained from the same experiment is presented in Movie S8 (*Online Supplementary Material*).

The impairment of excitation wave propagation and an OAPD distribution that may indicate the formation of an anatomical obstacle in the LV of the pig heart by OM *in situ* were obtained under artificial blood circulation using di-4-ANBDQBS. The formation of VF and reentrant arrhythmias around stable potential anatomical obstacles are shown in Fig. 6c,d (*see also Online Supplementary Material*, Fig. S7). The activation time and OAPD50 maps (Fig. 6c) were constructed from Movie S9 (*Online Supplementary Material)*. In the movie, the main excitation waves can be observed to circulate around the damaged tissue. The main circulation wave draws in the excitation waves from the left side of the LV. Both waves are shown by white arrows at the bottom of the activation map (Fig. 6c*, left column*). We can also see the secondary propagation waves of the excitation from the main sources propagating to the RV. These secondary waves generate multiple excitations throughout the heart. The OAPD50 maps (Fig. 6c*, right column*) show the notable differences in the OAPD that coincide with the sites of severe conduction impairment (see locations in the activation maps with the greatest crowding of isochrones).

## Discussion

Our study was designed to adapt the high-spatiotemporal-resolution OM technique widely used in isolated hearts to the cardiac imaging of pig hearts *in situ*. The registration of cardiac OAPs using di-4-ANBDQBS or Cardiogreen was performed under physiological or artificial blood circulation. The low quality of electrical activity recordings under physiological blood circulation encouraged us to develop an OM system for the pig heart *in situ* under artificial blood perfusion, a condition that is widely used in cardiac surgery. Overall, the results confirm that cardiac electrical activity could be optically mapped *in situ* under normal or arrhythmic conditions in a large animal model.

Previously, electrical mapping of the heart *in situ*, including in humans, was performed using multiple arrays of electrodes^4,13^. However, OM using voltage-sensitive dyes has a great advantage over electrical mapping in that the spatiotemporal resolution is much higher. The dual OM of voltage and calcium in small mammalian hearts *in situ* has been reported^12^. Recently, a paper in which OM was used in a pig model *in vivo* was published. The OS from di-4-ANBDQBS was recorded in the contracting heart. At physiological contraction rates, the OAPs were recorded using a 2D fibre array from 16 locations of the heart and ratiometry^17^. At first glance, our work may seem to present similar data as the recent publication by Lee *et al*.^17^. However, in principle, Lee and co-authors present two methods for recording cardiac electrical activity in an *in vivo* pig model: excitation ratiometry in a freely contracting heart and optical fibre recordings from 16 points. In our study, we present two different modes for OM in an *in situ* pig model: mechanical immobilization and the chemical arrest of heart contractions. In our opinion, cardiac OM is as interesting as it allows the recording of electrical activity at both a high spatial and temporal resolution. The strength of OM is that it allows the construction of various maps, such as maps of the activation time, AP duration and other parameters, across the entire heart surface. Lee *et al*.^17^ presented activation maps recorded in both modes (excitation ratiometry and optical fibre recordings). However, the OAPD maps were constructed using only optical fibre recordings from 16 points in an area of 12 × 12 mm of the heart, and no OAPD maps from mapping of the whole heart using excitation ratiometry were presented. Considering that the anterior surface of the pig heart is at least 50 × 50 mm in size, obviously, recordings from an area of 12 × 12 mm cannot provide detailed information about the propagation of electrical signals in the heart and cannot be considered to provide high-spatial-resolution mapping. Our presented data include activation time, OAPD, repolarization time, and conduction velocity maps constructed from OSs obtained from a large mapping area, i.e., 55 × 55 mm, under artificial blood circulation. Additionally, other disadvantages of the ratiometric analysis used in the study by Lee *et al*.^17^ could be emphasized, such as its limited potential for application in freely contracting hearts. Excitation ratiometry can reduce the influence of heart contraction movements, but it cannot eliminate the effects of the heart twisting without any immobilization, which is always present in freely contracting hearts. It has been demonstrated that to eliminate the effects of twisting, heart immobilization or mathematical data processing should be used^6,18^.

The open-chest pig heart model can be used in two ways: with and without an artificial blood circulation system. Under physiological blood circulation, heart contractions are obligatory, while under artificial blood circulation, the heart can be fully stopped.

To use voltage-sensitive dyes for the registration of electrical activity, the pixel registered by the camera and the exact site of tissue registration should be aligned^19^. Chemical and mechanical immobilization and online/offline computational methods are used to create suitable conditions for OM to reduce the influence of heart motion on the OSs (for a review, see^29^).

Heart contractions are essential for supplying the organism with oxygen and removing metabolites, and the chemical blocking of contractions is generally unusable. Therefore, during experiments performed under physiological blood circulation, we mechanically immobilized part of the heart. Our experiments show that the optical registration of cardiac electrical activity *in situ* was successful because the obtained signals were sufficient to register electrical behaviour throughout almost the entire mapping area. However, the OS was not ideal, since contraction artefacts still occurred. This phenomenon may restrict the available voltage-sensitive dye options because under such conditions, only dyes (preferably NIR dyes) with high fractional fluorescence could be used. Notwithstanding, the use of a frame or cardiac surgical tissue stabilizer enables the registration of optical cardiac electrical activity *in situ* under normal conditions or VT/VF. The main disadvantage of mechanical motion arrest using a frame or tissue stabilizer is that the field of view is quite limited; therefore, the technique may not be suitable for the investigation of certain patho/physiological conditions.

Our results show that standard artificial blood circulation provides controlled conditions and that under such conditions, OM can be applied to record the electrical activity of the heart in large animals *in situ*. Under artificial blood circulation, heart contractions are not necessary for blood circulation; therefore, activity of the heart can be fully stopped. OM under artificial blood circulation approximates the conditions under Langendorff perfusion of the isolated heart because under both conditions, the heart is perfused in a retrograde manner through the aorta. Artificial blood circulation enables the use of chemical uncouplers and the performance of experiments *in situ* without excision of the heart from the organism, which may change the activity of the heart^7^. Moreover, OM *in situ* allows evaluation of the influence of the autonomic nervous system as well as humoural regulation on the electrical activity of the heart. The chemical arrest of contractions allowed the use of the fluorescent dye Cardiogreen, whose ΔF/F is markedly lower than that of di-4-ANBDQBS. The results supplemented those of our previous report on the voltage sensitivity of Cardiogreen^10^. However, the data on the effect of blebbistatin on cardiac electrical activity are controversial^17,24,25^. Our results show that under artificial blood circulation, the one-time bolus containing blebbistatin did not have a significant effect on the electrogram of the pig heart *in situ*. Our data are in agreement with those of recent studies, which did not observe obvious electrophysiological changes following blebbistatin perfusion in the intact guinea pig heart^14^ or pig heart under Langendorff perfusion^6^. Notwithstanding, the lack of comprehensive investigations of the cardiotoxicity of this uncoupler is still a limiting factor for the application of OM in pre-clinical/clinical studies.

The use of voltage-sensitive dyes in OM *in situ* means that the dyes enter the blood environment; therefore, only NIR dyes can be used effectively to match the haemoglobin optical window^8^. As these dyes are excited by long wavelengths of light, the emitted fluorescence originates from a considerable tissue depth. This behaviour is of particular value in the OM of large mammalian hearts, which have thick ventricular walls and massive coronary vessels. Although the voltage-sensitive dyes used in this study require long excitation wavelengths, our results show that the anatomical site of the main subepicardial coronary vessels located in the anterior interventricular sulcus distorts the OAPs. This result is consistent with data from a simulation approach demonstrating that the OAP upstroke is prolonged near large subepicardial vessels or shows a distinct “humped” morphology^30^.

Thus, our study is the first to apply high-spatiotemporal-resolution OM in the pig heart *in situ* to record cardiac electrical activity qualitatively under artificial blood perfusion. The use of an FDA-approved, voltage-sensitive dye and artificial blood perfusion in a swine model, which is generally accepted as a valuable pre-clinical model, demonstrates the promise of OM for clinical application.

## Methods

Experiments were performed in an open-chest pig heart model under physiological or artificial blood circulation. All experiments were performed according to the European Community guiding principles and approved by the State Food and Veterinary Service of the Republic of Lithuania (No. G2-68, 21 June 2017).

Twenty pigs weighing 35–40 kg were premedicated intramuscularly with atropine sulphate (0.05 mg/kg, Sanitas AB, Lithuania), xylazine hydrochloride (4 mg/kg, Bela-Pharm GmBH and Co., KG, Germany) and ketamine hydrochloride (30 mg/kg, Richter Pharma AG, Austria). An Edan IM70 data monitor (Edan Instruments, China) was then connected to the tail to monitor the status of the anaesthetised animal. Under sterile conditions, an 18-G catheter (Provein, Lars Medicare, Haryana, India) was inserted into the lateral auricular vein. A three-way connector (B-Braun Melsungen AG, Germany) and infusion system (B-Braun Melsungen AG, Germany) were connected, and the administration of 0.9% sodium chloride saline (Fresenius Kabi, Poland) was started. The pigs received 25,000 IU of heparin sodium (Rotexmedica GmBH, Germany) intravenously to prevent blood clotting.

### Induction and maintenance of anaesthesia

Thiopental sodium (10 mg/kg, Sandoz International GmBH, Germany) was injected intravenously to stop breathing. Then, a ventilation apparatus (Harvard Large Animal Ventilator, 613; 115 VAC; Canada) for artificial lung ventilation was connected. A detailed description of the intubation procedure is provided in the *Online Supplementary Material*. Animals were subjected to long-term, deep anaesthesia by the intravenous injection of propofol (5–6 mg/kg/9 min, Norameda UAB, Lithuania) by a Harvard Apparatus infusion pump (Pump 11, Pico plus Elite, USA). Opioids were used for analgesia, and butorphanol tartrate (2 mg/kg, Richter Pharma AG, Austria) was administered every hour. Myorelaxation was provided intravenously using pipecuronium bromide (0.1 mg/kg, Gedeon Richter, Hungary), which was repeated every hour during surgery. Before the thoracic cavity incisions, local subcutaneous anaesthesia was established with lidocaine (20 mL, Sanitas AB, Lithuania) solution (20 mg/mL).

Electrocardiography, pulse, and arterial blood pressure monitoring was performed by an Edan IM70 device (Edan Instruments, China) throughout the experiment. The arterial blood gas and electrolyte levels were measured as well. All parameters were maintained within normal ranges throughout the study.

After the experiments, the pigs were euthanized intravenously with pentobarbital sodium (7–10 mL, Dolethal, Vetoquinol, France).

### Artificial blood circulation

The animals were subjected to the cardiopulmonary bypass system, to achieve full haemodinamic stability and conditions similar to open heart surgery (on pump approach). Cardiopulmonary bypass was established by cannulating the distal ascending aorta. An aortic root cannula with a vent line was introduced into the ascending aorta below the aortic cannula for the delivery of dye solutions into the coronary circulation. After establishment of the cardiopulmonary bypass system, artificial lung ventilation was stopped.

All surgical techniques and artificial blood circulation connections are presented in detail in the *Online Supplementary Material*.

### Motion arrest

During open-heart surgery under physiological blood circulation and lung ventilation, two techniques were applied. First, a hand-made metal frame was used as an alternative for the mechanical contraction arrest of the heart to immobilize an area of the heart surface (Fig. 1c). The metal frame (with a window of 40 × 40 mm) was fixed onto the heart. Problems occurred when fixing the frame directly onto the beating heart. For this reason, Velcro tape was used, with one side fixed to the heart surface with clinically used cyanoacrylate glue and the other fixed to the metal frame. After adhering both sides of the tape, the heart was slightly lifted using an additional bar (indicated by arrow in Fig. 1c) which was fixed to the manipulator. In this way, the influence of motion caused by lung ventilation was minimized. Using metal frame, the posterior part of the heart could freely contract and ensure normal blood circulation while contraction (or at least twisting) of the anterior part of the heart was immobilized.

In some experiments, an Octopus tissue stabilizer was used (Octopus 4.3, Medtronic, USA) (Fig. 1d). This device is commonly used in beating heart procedures in cardiac surgery to achieve the local suspension of heart contractions in a certain area and avoid artificial blood circulation. The tissue stabilizer was connected to a vacuum pump (max. vacuum level, 85 kPa; VMECA, South Korea), and suction was applied to the heart surface around the expected mapping area. The heart was gently lifted under vacuum suction to overcome the motion caused by lung ventilation.

In experiments with artificial blood circulation, which allows the use of chemical uncouplers, heart motion was eliminated by the injection of a 100-mL bolus containing 600 µmol/L blebbistatin together with fluorescent dye directly into a cannula inserted into the aorta. The final concentration of blebbistatin in the animal blood (~4000 mL) was 15 µmol/L.

### Staining

Two fluorescent dyes were used: the NIR fluorescent, voltage-sensitive dye di-4-ANBDQBS and indocyanine green (Cardiogreen), which also has voltage-sensitive properties^9–11^. The dyes were injected through the aortic root cannula, and the vent line was inserted into the aorta. To obtain maximal staining of the heart tissue, during the dye injection the aorta was clamped below the arterial return cannula. The dye injection lasted no more than 2 min. Other details of the staining procedure differed depending on the blood circulation model.

Under physiological blood circulation (n = 7), staining was performed under pressure control to obtain sufficient delivery of the dye into the coronary circulation to avoid dye leakage in the left ventricular chamber. A 300-mL Ringer acetate Fresenius infusion saline (Fresenius Kabi; the composition of the saline is presented in *Online Supplementary Material*) bolus containing 20 µmol/L di-4-ANBDQBS was injected using a peristaltic pump, and the perfusion velocity was gradually increased to obtain the appropriate pressure. The pressure was monitored by a monometer attached to the tube connected to the aorta catheter. The injection was performed under pressure starting at 80 mmHg and gradually increasing to a maximum of ~140 mmHg until the staining became apparent by monitoring of the heart with an EMCCD camera. The visualization of the coronary arteries with the NIR fluorescent, voltage-sensitive dye is described and presented in the *Online Supplementary Material* (Fig. S1).

Under artificial blood circulation (n = 13), a 100-mL Ringer acetate Fresenius infusion saline bolus containing 50 µmol/L di-4-ANBDQBS (n = 8) or 120 µmol/L Cardiogreen (n = 5) and blebbistatin was injected directly through the infusion system. The di-4-ANBDQBS and Cardiogreen loading process is presented in Movies S10 and S11, respectively (*Online Supplementary Material*).

Under both experimental conditions, the final concentration of di-4-ANBDQBS in the circulating blood (~4000 mL) was ~1.5 µmol/L, and the final concentration of Cardiogreen was ~3 µmol/L.

Physiological saline was always heated to +38 °C before use. Five millilitres of an aqueous stock solution of Cardiogreen (2 mg/mL) was freshly prepared before every experiment.

### Optical mapping

The OM setup consisted of excitation sources, emission filters, and a high-speed camera, and it was adapted for the registration of cardiac electrical activity in the pig heart *in situ* during open-heart surgery (Fig. 1). Di-4-ANBDQBS was excited with red light by a 671-nm diode laser (MRL-N-671-3W, Roithner LaserTechnik, Austria) equipped with a diffuser to increase the area of excitation and four collimated 660-nm LEDs (Thorlabs, USA) filtered at wavelengths of 650 ± 20 nm with a bandpass filter. Using both excitation sources together ensured higher-intensity and more homogenous di-4-ANBDQBS signals from the large mapping area. The emission was measured using a 720-nm long-pass filter (NT46-066, Edmund Optics, USA). For fluorescence ratiometry, OSs were also recorded additionally with green light excitation at 532 nm using a diode laser (MGL-F-532-3W, Roithner LaserTechnik, Austria), and the emissions were collected using a 630/69-nm bandpass filter (Semrock). Under such conditions, the OAPs had positive polarity and presented values opposite to those of OAPs with negative polarity at λ_ex_ = 660 nm/λ_em_ = 720 nm.

Cardiogreen was excited by four collimated LEDs (Thorlabs, USA) at a wavelength of either 660 nm or 780 nm (filtered at 650 ± 20 nm and 780 ± 5 nm, respectively). Altogether, eight excitation LEDs were placed in a stable wheel (Fig. 1e). Cardiogreen fluorescence signals were collected with a bandpass filter (775 ± 23 nm) and long-pass filters (720 nm and 808 nm). The emission filters were mounted in a motorized filter wheel (8MRU-1WA; Standa, Ltd., Lithuania) that was positioned in front of the camera.

Optical movies were obtained with a cooled (−100 °C), fast, 14-bit EMCCD camera (iXonEM+DU-860, Andor Technology, Ireland) equipped with a 50-mm focal length with F/0.95 high speed objective MVL50HS (Navitar, USA). Based on study of Bien *et al*.^31^, Navitar lenses with such parameters are one of the best choices available for OM of the heart of big animal. The anterior surface of the heart, including the RV, anterior interventricular sulcus, and LV, was imaged. The overall mapping area was 55 × 55 mm. When the frame was used, the effective mapping area was 40 × 40 mm. Using imaging software (Andor SOLIS x-3467), optical movies were acquired at a frame rate of 500 Hz with a resolution of 128 × 128 pixels. One pixel corresponds to 0.43 mm square on the heart surface. For optical recordings, the heart was paced via bipolar electrodes with 2-ms stimuli at twice the diastolic threshold. Bipolar hook electrodes were embedded into the atrium, ventricle or septum.

### Processing of optical data

The optical movies were processed using ImageJ software. The OAPs presented in the figures were taken from an area of 5 × 5 pixels. The background fluorescence (F) was subtracted from every frame of the recording. The OS was normalized with respect to the background fluorescence to obtain the fractional change in the fluorescence signal (ΔF/F). The OAP maps were constructed using custom Scroll 1.16 software developed by Dr. S. Mironov (University of Michigan). Before creating the maps, the OSs were additionally filtered using three-point triangular time and 5 × 5 pyramidal kernel space filters. The activation time maps show the time interval from stimulus to 50% depolarization of the OAP. The OAPD50 and OAPD80 maps show the duration of the OAP at the level of 50% and 80% repolarization from activation time, respectively. The repolarization time maps show the OAPD50 along with the activation time.

The VT/VF dominant frequency (DF) was obtained from the frequency spectrum of the OSs using a Fourier transform.

### Statistics

The significance of the difference between various parameters of OSs recorded from different dyes under normal or artificial blood circulation was evaluated using one-way ANOVA. A value of p < 0.05 was considered statistically significant.

## Supplementary information

Online Supplementary Material.

Movie S1.

Movie S2.

Movie S3.

Movie S4.

Movie S5.

Movie S6.

Movie S7.

Movie S8.

Movie S9.

Movie S10.

Movie S11.
