# Supplementary material for: Optical mapping of the pig heart in situ under artificial blood circulation
Source: Sci Rep. 2020 May 22;10:8548. doi: 10.1038/s41598-020-65464-5 (PMC7244500; doi:10.1038/s41598-020-65464-5)
Supplement: Supplementary file 1 — Online Supplementary Material. [file 41598_2020_65464_MOESM1_ESM.docx]

# Optical mapping of the pig heart *in situ* under artificial blood circulation

**Supplementary material**

# Irma Martišienė^1^, Dainius Karčiauskas^1,2^, Antanas Navalinskas^1^, Regina Mačianskienė^1^, Audrius Kučinskas^1,3^, Rimantas Treinys^1^, Ramunė Grigalevičiūtė^1,3^, Vilma Zigmantaitė^1,3^, Laima Ralienė^2^, Rimantas Benetis^1,2^, Jonas Jurevičius^1^*

^1^Institute of Cardiology, Lithuanian University of Health Sciences, Kaunas, Lithuania.

^2^Department of Cardiac, Thoracic and Vascular Surgery, Hospital of Lithuanian University of Health Sciences Kauno Klinikos, Lithuanian University of Health Sciences, Kaunas, Lithuania.

^3^Biological Research Centre, Lithuanian University of Health Sciences, Kaunas, Lithuania.

*Corresponding author. Sukilėlių 15, LT-50162 Kaunas, Lithuania. Tel: +370-37-302877, Fax: +370-37-220733, Email: jonas.jurevicius@lsmuni.lt

**This file includes the following:**

Supplementary Results, Including Supplementary Figures S1 to S7

Supplementary Methods

Supplementary References

Legends for Supplementary Movies S1 to S11

**Other Supplementary material for this manuscript includes the following:**

Supplementary Movies S1 to S11

**Supplementary Results**

**Visualization of the coronary arteries with NIR fluorescent, voltage-sensitive dyes**

The near-infrared (NIR) fluorescent dye Cardiogreen is widely used in clinical practice to visualize vessels in various organs (Handa et al., 2009; Alander et al., 2012). To determine the potential of the NIR voltage-sensitive dyes we used for visualizing coronary vessels and to observe the dye loading process, we took images beginning during the initial seconds of the staining procedure. The detailed process of loading the heart tissue with the fluorescent dyes di-4-ANBDQBS and Cardiogreen *in situ* is presented in images obtained by an EMCCD camera at different time points during the injection of the solution containing the dye (*Figure S1*). At the start of the staining procedure, when the stream of blood from the aorta is temporarily stopped, the coronary arteries are visible, indicating that the solution containing the dye flows first through the arteries (*Figure S1a* and *d*) and then through the veins. At this stage, the heart tissue is also already stained (*Figure S1b* and *e*). Finally, images obtained after a few minutes, when the aorta is unclamped below the arterial return cannula, show that the dye is distributed evenly throughout the heart tissue and that the coronary vessels are not clearly visible because of recovered blood perfusion (*Figure S1c* and *f*). The results show that the process of staining was analogous for both dyes, i.e., di-4-ANBDQBS and Cardiogreen. Entirely stained heart tissue prevents visualization of the coronary vessels, suggesting that this benefit of NIR voltage-sensitive dyes in such conditions is transient.

Thus, the images clearly show that in addition to voltage sensitivity, these fluorescent dyes have the benefit of visualizing the coronary arteries because the dyes stain the blood cells first. This result suggests that the use of NIR voltage-sensitive dyes for the investigation of electrical activity could also enable transient visualization of the quality of flow through the coronary arteries.


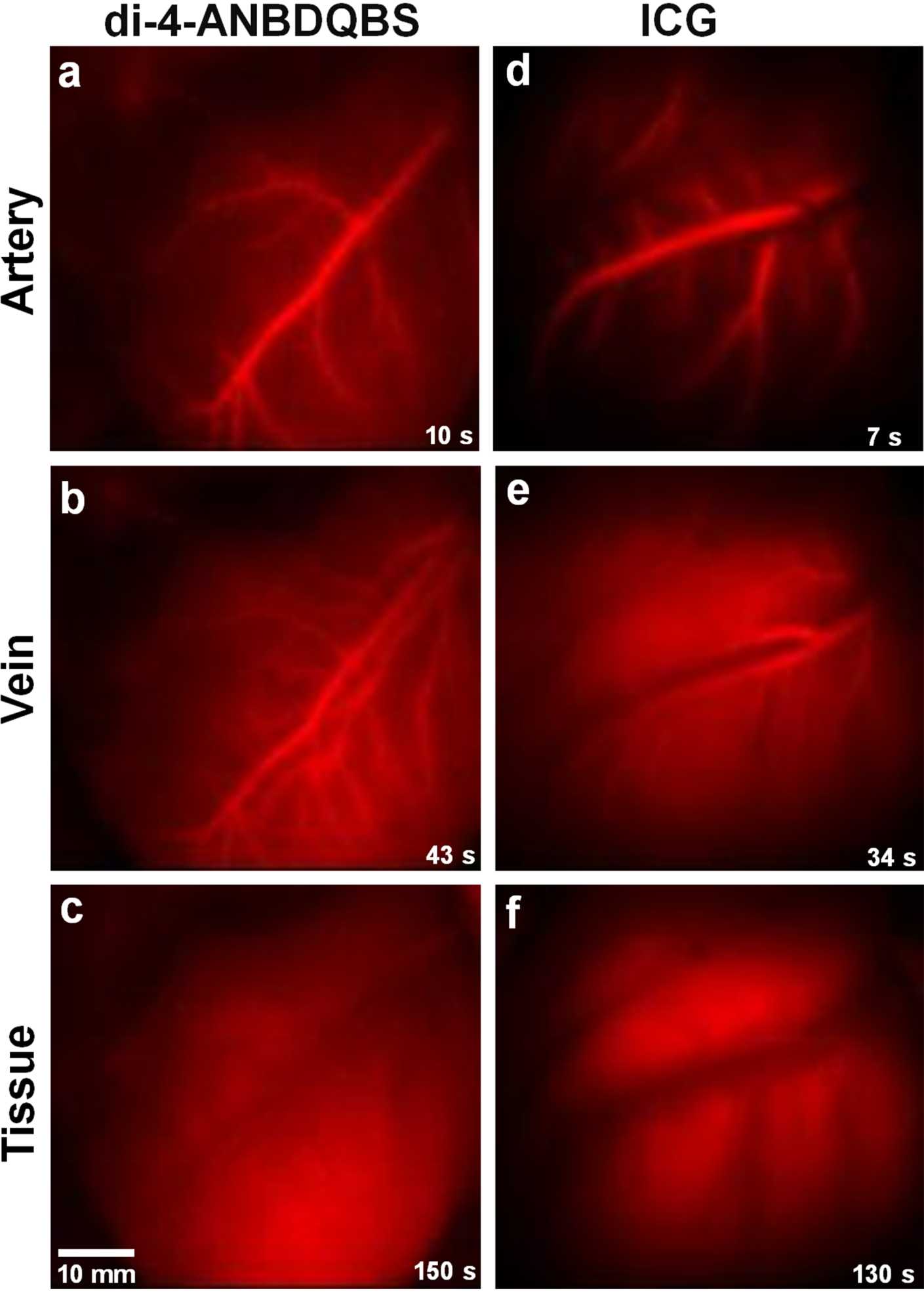


**Figure S1.** Representative images of the pig heart at different times during the loading of the fluorescent dyes di-4-ANBDQBS (*a-c*) and Cardiogreen (*d-f*). Loading of the heart arteries (*a,d*), veins (*b, e*), and tissue (*c,f*) with voltage-sensitive dyes. Seconds in each image indicate the time after the dye injection. The mapping area was 55x55 mm. Red-scale colour was applied.

**Optical mapping of pig heart *in situ* using fluorescent di-4-ANBDQBS dye**

**
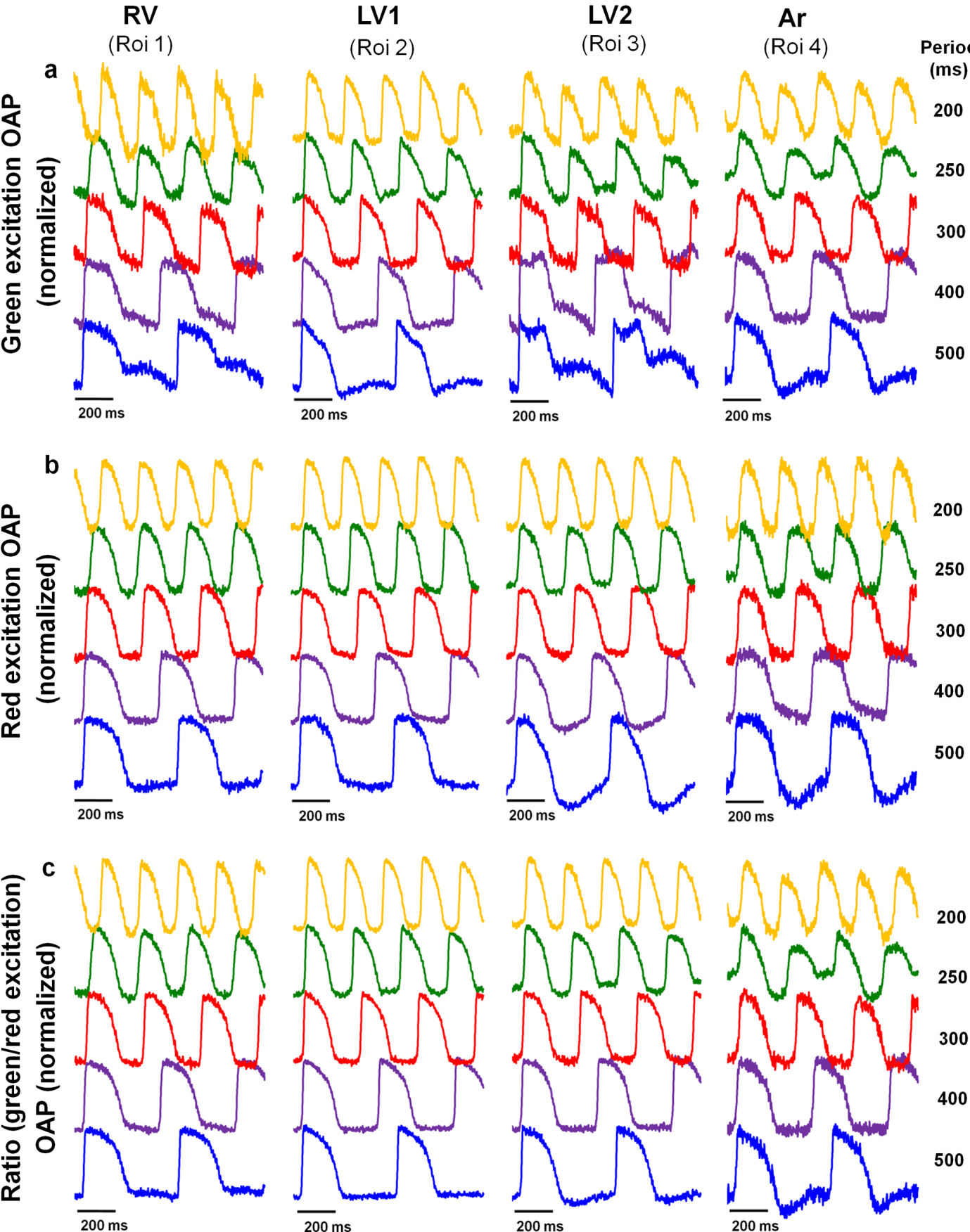
**

**Figure S2.** Normalized optical action potentials (OAPs) in the pig heart recorded by optical mapping *in situ* using di-4-ANBDQBS under artificial blood circulation at different stimulation periods (200-500 ms) and excitation wavelengths (green, red). OAP examples are taken from several heart locations: right ventricle (RV), left ventricle (LV), and coronary artery (Ar). The exact locations (ROIs 1-4, 5×5 pixels) from which the OAPs were averaged are indicated in *Figure S3*. (*a*) OAPs obtained under green excitation. (*b*) OAPs obtained under red excitation and OAPs with inverted polarity. (*c*) OAPs obtained by the ratio of signals under green and red excitation.

**
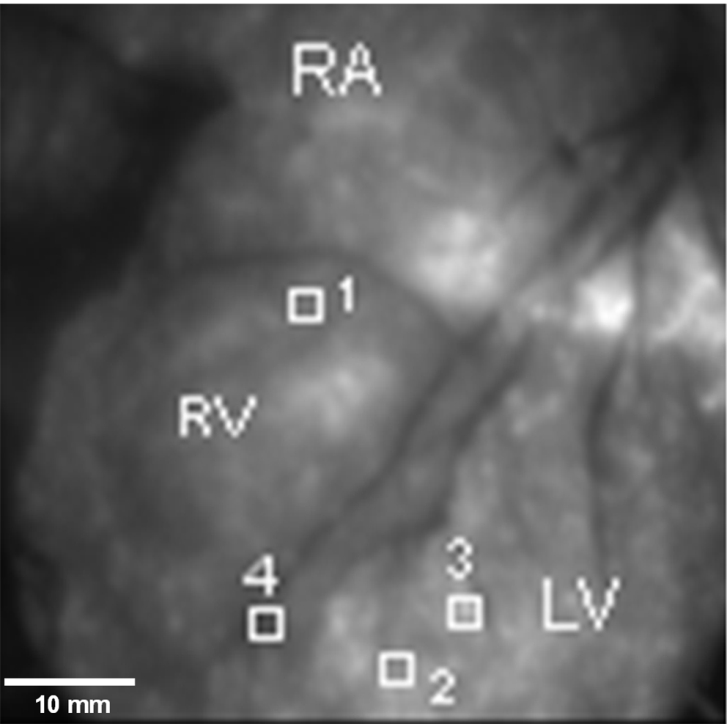
**

**Figure S3.** Image of the mapping area (55x55 mm) of the pig heart. RA, right atrium; RV, right ventricle; LV, left ventricle. Numbered squares indicate the exact locations of the 5x5-pixel ROIs from which the optical action potentials were averaged, as shown in Figure S2.


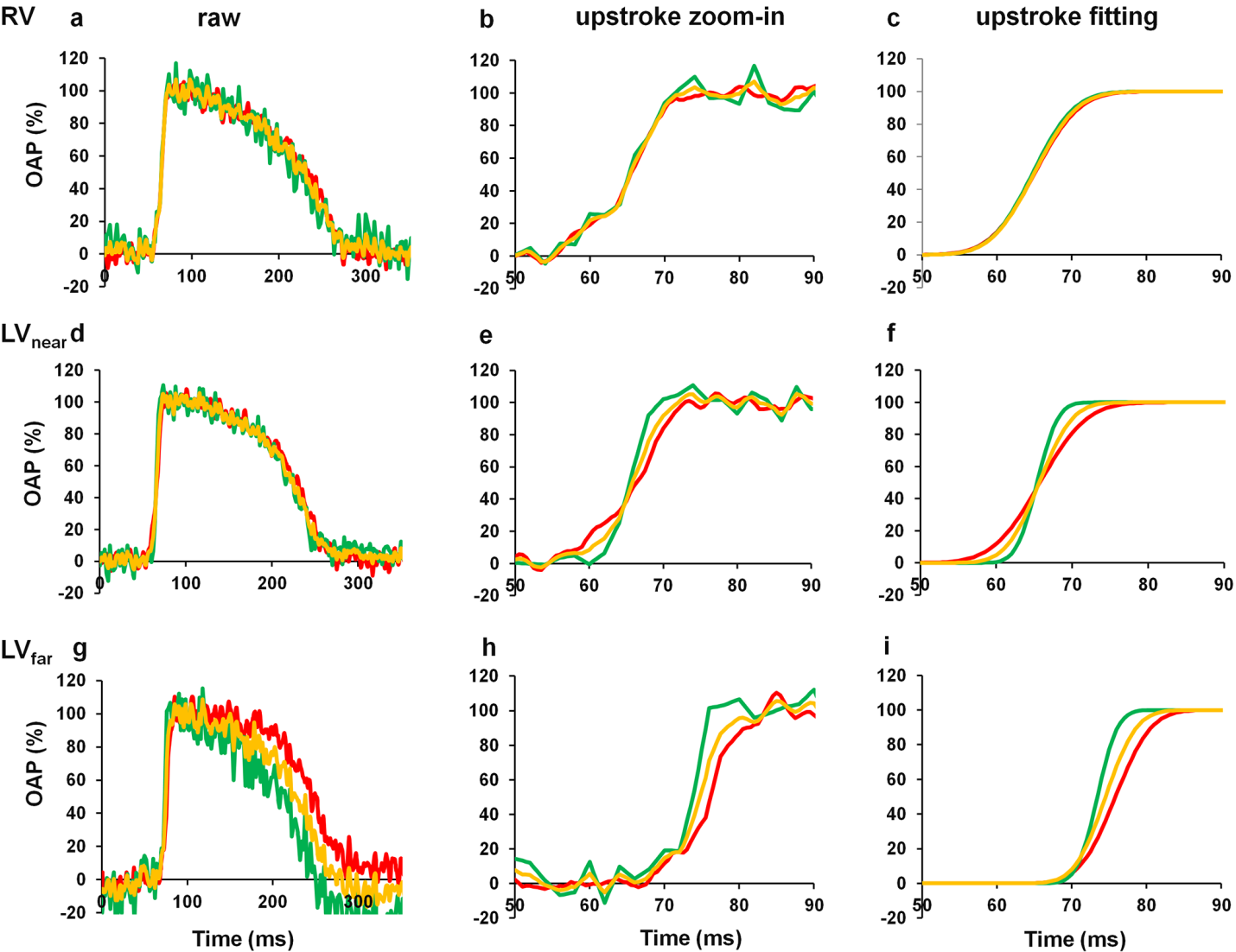


**Figure S4.** Optical action potentials (OAPs) in the pig heart recorded by optical mapping *in situ* using the fluorescent dye di-4-ANBDQBS under artificial blood circulation during pacing with a 500-ms period. OAPs obtained at λ_ex_=532 nm (green curves) and at λ_ex_=660 nm (red curves), and their ratio (yellow curves), are shown at normal and magnified time scales. The left and middle columns show the raw OAPs and upstrokes, and the right column shows the upstrokes fitted with the double sigmoidal function. *a-c* show the OAPs of the right ventricle, and *d-f* and *g-i* show the OAPs of the left ventricle close to and far from the stimulation location, respectively.

**OAPD80 of pig heart recorded by optical mapping *in situ***


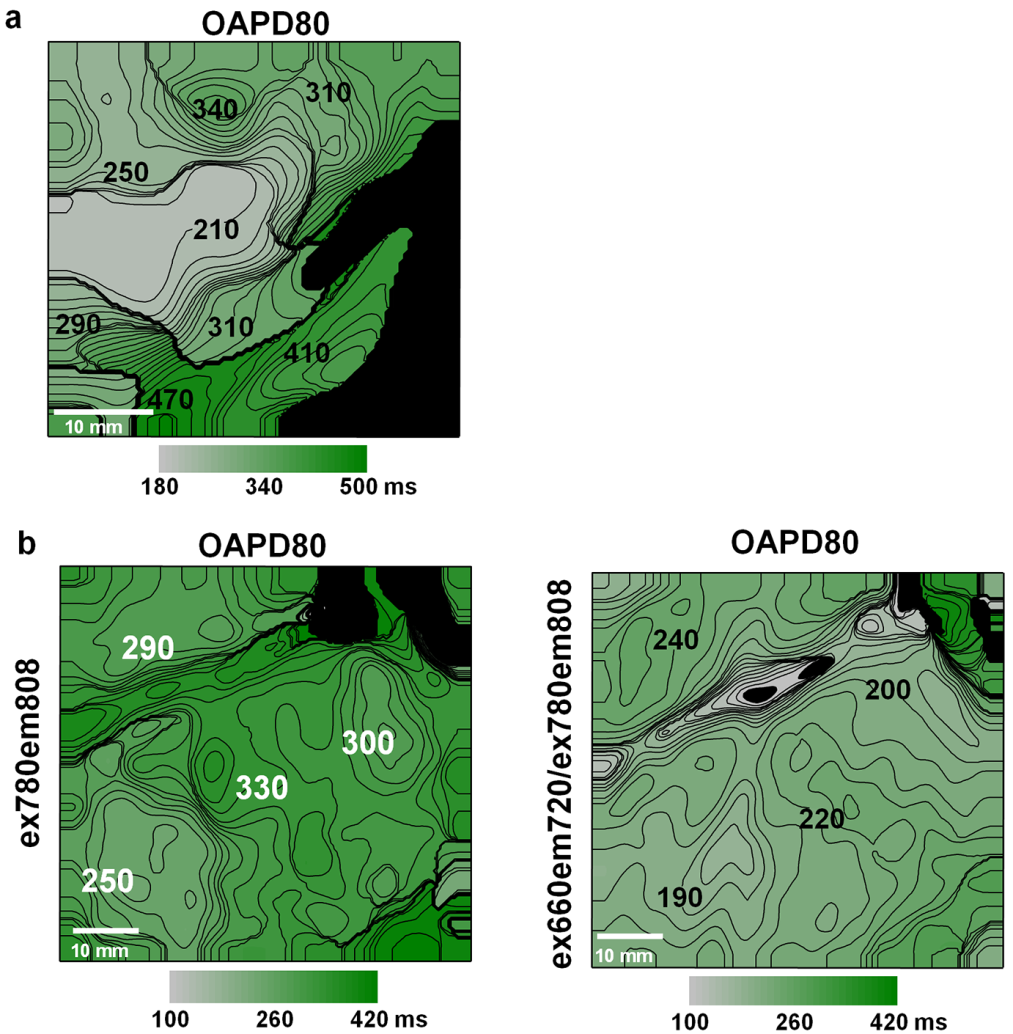


**Figure S5.** OAPD80 of pig heart recorded by optical mapping *in situ.* (*a*) OAPD80 map obtained using the fluorescent dye di-4-ANBDQBS under physiological blood circulation. (*b*) OAPD80 maps obtained using the fluorescent dye Cardiogreen under artificial blood circulation. *Left,* maps obtained at λ_ex_=780 nm and λ_em_=808 nm; *right,* maps constructed from the OAPs calculated as the ratio of the two OSs obtained at λ_ex_=660 nm/λ_em_=720 nm and λ_ex_=780 nm/λ_em_=808 nm.

**Optical mapping of the pig heart *in situ* using fluorescent, voltage-sensitive dyes in pathological situations**

**Regional ischaemia.** *Figure S6* shows representative recordings of the optical signals obtained during ventricular pacing with a 500-ms period under the control and regional ischaemia conditions. The maps of the activation time, OAPD50, repolarization time and OAPD80 (*Figure S6a, b, c and d, respectively*) show the propagation wave and distribution of the APD, which depend on the part of the heart, i.e., the left ventricle (LV), right ventricle (RV), and coronary artery (Ar). Examples of OAPs from the sites marked on the activation maps are presented in *Figure S6e* and *f*.


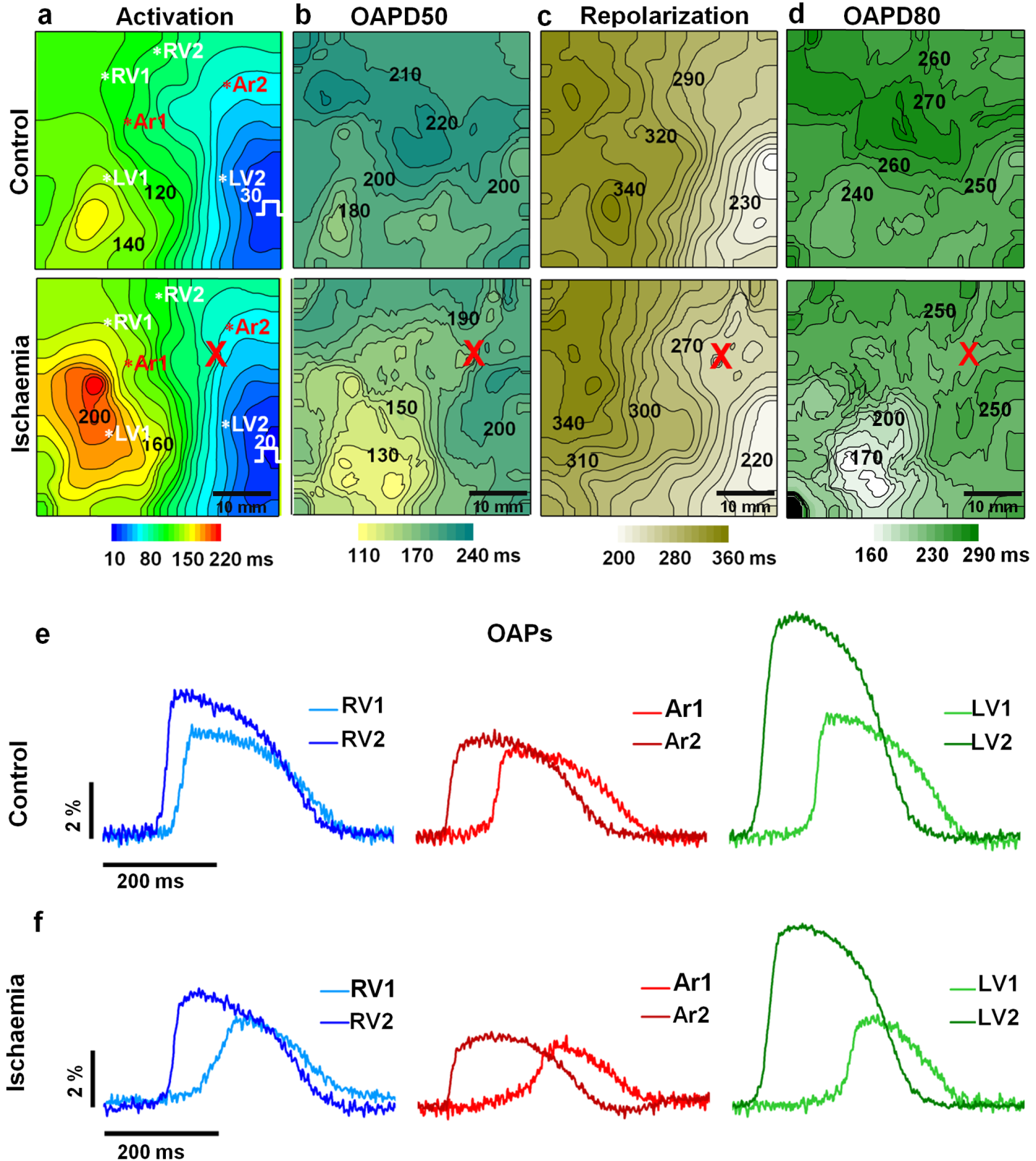


**Figure S6.** Electrical activity in the pig heart recorded by optical mapping *in situ* using the fluorescent dye di-4-ANBDQBS under artificial blood circulation in the control and ischaemia conditions. Ventricular pacing with a 500-ms period was applied. (*a*) Activation time maps. (*b*) Optical action potential duration at 50% repolarization (OAPD50) maps. (*c*) Repolarization maps. (*d*) Optical action potential duration at 80% repolarization (OAPD80) maps. X indicates the site of left anterior descending artery occlusion. The numbers near the isochrones show the activation time, OAPD50, OAPD80 and repolarization time in ms. The interval between isochrones is 10 ms. The OAPs from two different sites in the right ventricle (RV1, RV2), coronary artery (Ar1, Ar2) and left ventricle (LV1, LV2) (indicated in the activation maps) under the control and ischaemia conditions are presented in (*e*) and (*f*), respectively. The amplitude of the OAPs is presented as a percentage with respect to the background (ΔF/F). The different magnitude of the OS from the left and right ventricles may be related to various factors, such as dye loading, illumination, and non-excitable tissues (vessels, fat). The signals obtained from the region of the main coronary arteries are weaker because in such circumstances, the arteries form a morphological obstacle to the OS from the underlying heart tissue.

**Ventricular tachycardia/fibrillation.** The data presented in *Figure S7* were obtained under artificial blood circulation using di-4-ANBDQBS. In the given example, ventricular tachycardia (VT) was followed by the induction of regional ischaemia and reperfusion. The activation time maps constructed from three successive beats clearly illustrate the propagation around the stable anatomical obstacle, as indicated by a red circle (*Figure S7a-c*). The main direction of propagation was evaluated by eye and labelled with white arrows. Accordingly, the OAPD50 maps show the distribution of the OAPD *(Figure S7d-f*). The notable differences in the OAPD coincide with the sites of severe conduction impairment. The raw recordings of OAPs obtained at the sites of the RV and LV, indicated by asterisks in the activation maps, show the order of OAP activation (*Figure S7g*) starting from the vertical dashed line.


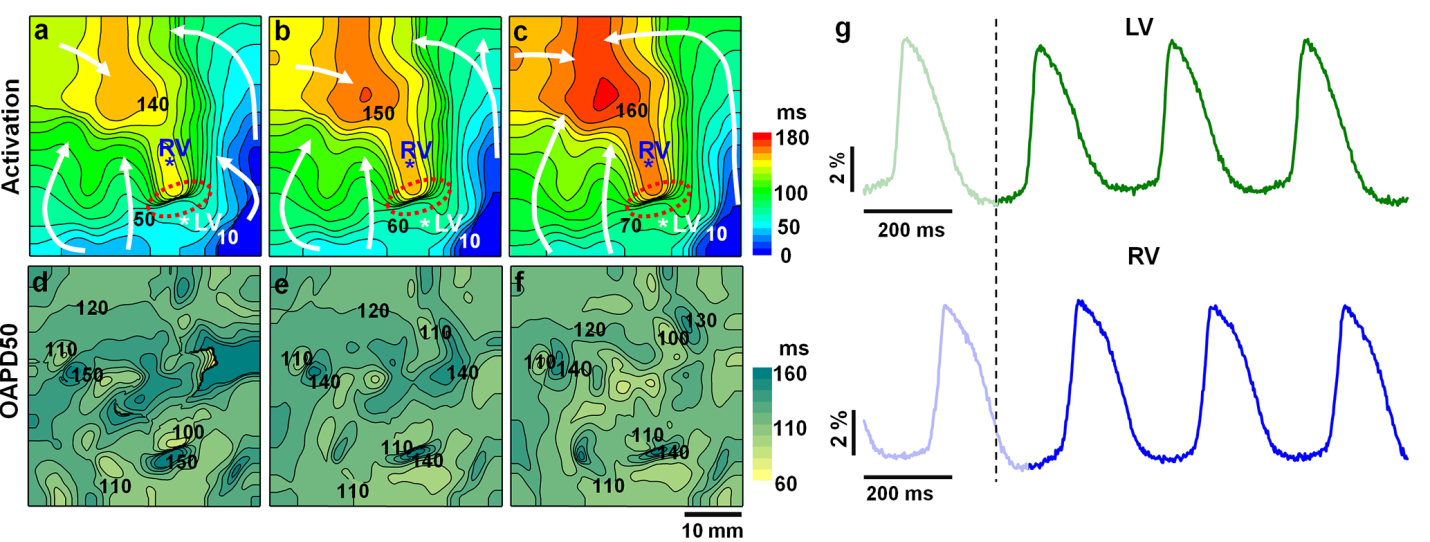


Figure S7. Impairment of excitation wave propagation and distribution of the OAPD indicating the formation of an anatomical obstacle in the LV of the optically mapped pig heart *in situ* under artificial blood circulation. (*a-f*) Activation time and OAPD50 maps. White arrows indicate the directions of wave propagation. The red dotted circle indicates the stable anatomical obstacle. The numbers near the isochrones show the activation time and OAPD50 in ms. The interval between isochrones is 10 ms. (*g*) Raw optical traces from the sites of the RV and LV, indicated by asterisks in the activation maps. The three bright OAPs (*g*) correspond to the three OAPD50 maps. The amplitudes of the OAPs are given as percentages with respect to the background (ΔF/F). The dominant frequency in the RV was 13.7 Hz.

**Supplementary Methods**

**Induction of artificial lung ventilation.** After thiopental sodium (10 mg/kg, Sandoz International GmBH, Germany) was injected, the pigs stopped breathing. The animals were positioned correctly, and roll bandages (soft rope or similar) were placed around the upper and lower jaws to facilitate opening the mouth and holding the head and neck elevated and extended. Using a laryngoscope, the larynx was visualized, and the mouth cavity was sprayed with 10% lidocaine spray solution (Egis Pharmaceutical, Hungary) to prevent instantaneous respiratory spasm during intubation when the laryngoscope touched the larynx. An endotracheal tube (ET) (Kruuse, Denmark) of an appropriate diameter was inserted and secured, and the cuff was periodically inflated. After introduction of the ET into the trachea, the tube was connected to the ventilation apparatus (Harvard Large Animal Ventilator, 613; 115 VAC; Canada) for artificial lung ventilation. The pigs were ventilated at 20 BPM. Both manual and mechanical monitoring was performed. The eye position, palpebral reflex, mucous membrane colour and capillary refill time were monitored manually.

**Surgical techniques and artificial blood circulation.** Surgery was performed via a median sternotomy and opening of the pericardium. Thirteen experiments were performed under artificial blood circulation conditions. After the injection of heparin, cardiopulmonary bypass was established by cannulating the distal ascending aorta (18Fr cannula, Medtronic, Minneapolis, USA) and inserting a venous drainage line using two-stage right atrial cannulation (23French, Medtronic, Minneapolis, USA). An aortic root cannula with a vent line (Y-shaped, 12 Gauge, Minneapolis, USA) was introduced into the ascending aorta below the aortic cannula for the delivery of dye solutions into the coronary circulation. Two COBE (Lakewood, Co., USA) peristaltic pumps were used to drain the blood from the surgical area and circulate it through a heated (+38 °C) membrane oxygenator (Soring Group, Italy). The oxygenator was filled with 1000 mL of Ringer acetate Fresenius infusion saline (composition in mmol/L: 131 Na^+^, 4 K^+^, 2 Ca^2+^, 1 Mg^2+^, 111 Cl^-^, 30 acetate; Fresenius Kabi). After establishment of the cardiopulmonary bypass system, artificial lung ventilation was stopped.

**Supplementary References**

Handa T, Katare RG, Sasaguri S, Sato T. Preliminary experience for the evaluation of the intraoperative graft patency with real color charge-coupled device camera system: an advanced device for simultaneous capturing of color and near-infrared images during coronary artery bypass graft. *Interact. Cardiovasc. Thorac. Surg.* **9**:150-154 (2009).

Alander JT, Kaartinen I, Laakso A, Pätilä T, Spillmann T, Tuchin VV, Venermo M, Välisuo P. A review of indocyanine green fluorescent imaging in surgery. *Int. J. Biomed. Imaging* **2012**:940585 (2012).

**Supplementary Movie legends**

**Movie S1** Propagation of excitation in the pig heart recorded by optical mapping *in situ* using the fluorescent dye di-4-ANBDQBS over a 500-ms period of stimulation under physiological blood circulation. For mechanical heart immobilization, a hand-made frame was used.

**Movie S2** Propagation of excitation in the pig heart recorded by optical mapping *in situ* using the fluorescent dye di-4-ANBDQBS over a 500-ms period of stimulation under artificial blood circulation. The movie on the left (green-scale colour) shows the electrical activity recorded at λ_ex_=532 nm/λ_em_=640 nm, the movie in the middle (red-scale colour) shows the electrical activity recorded at λ_ex_=660 nm/λ_em_=720 nm, and the movie on the right (blue-scale colour) shows the ratiometric electrical activity.

**Movie S3** Propagation of excitation in the pig heart recorded by optical mapping *in situ* using the fluorescent dye di-4-ANBDQBS over a 400-ms period of stimulation under artificial blood circulation. Other notations are the same as in Movie S2.

**Movie S4** Propagation of excitation in the pig heart recorded by optical mapping *in situ* using the fluorescent dye di-4-ANBDQBS over a 300-ms period of stimulation under artificial blood circulation. Other notations are the same as in Movie S2.

**Movie S5** Propagation of excitation in the pig heart recorded by optical mapping *in situ* using the fluorescent dye di-4-ANBDQBS over a 250-ms period of stimulation under artificial blood circulation. Other notations are the same as in Movie S2.

**Movie S6** Propagation of excitation in the pig heart recorded by optical mapping *in situ* using the fluorescent dye di-4-ANBDQBS over a 200-ms period of stimulation under artificial blood circulation. Other notations are the same as in Movie S2.

**Movie S7** Propagation of excitation signals in the pig heart recorded by optical mapping *in situ* using the fluorescent dye Cardiogreen over a 500-ms period of stimulation under artificial blood circulation. This movie shows ratiometric electrical activity based on the ratio of optical signals recorded at λ_ex_=660 nm/λ_em_=720 nm and λ_ex_=780 nm/λ_em_=808 nm.

**Movie S8** Possible intramural propagation of the excitation wave recorded by optical mapping of the pig heart *in situ*. The fluorescent dye di-4-ANBDQBS was used. 3D view constructed using the ImageJ surface plot function. Resting to excited states are indicated by colours from blue to red. The X and Y axes show the mapping area in pixels, and the Z axis shows ΔF/F (%).

Movie S9 Reentry with a possibly stable anatomical obstacle recorded by optical mapping of the pig heart *in situ*. The fluorescent dye di-4-ANBDQBS was used. The main excitation waves circulate around the damaged tissue. The main circulation wave originates from the right side and draws in excitation waves from the left side of the left ventricle. Resting to excited states are indicated by colours from blue to red.

Movie S10 Di-4-ANBDQBS loading of pig heart *in situ* under artificial blood circulation.

Movie S11 Cardiogreen loading of pig heart *in situ* under artificial blood circulation.
